# Volumetric changes in the upper airway on CBCT after dentofacial orthopedic interventions - a systematic review

**DOI:** 10.1007/s00784-023-05207-8

**Published:** 2023-09-19

**Authors:** Ralph M. Steegman, Anne-Marie Renkema, Adriaan Schoeman, Anne Marie Kuijpers-Jagtman, Yijin Ren

**Affiliations:** 1 Department of Orthodontics, University of Groningen, University Medical Center Groningen, BB72 300001, Hanzeplein 1, Groningen, 9700RB The Netherlands; 2 Zijlweg Orthodontie, Orthodontic Private Practice, Haarlem, The Netherlands; 3 Department of Orthodontics, University Medical Center Groningen, University of Groningen, 9713 GZ Groningen, The Netherlands; 4 Department of Orthodontics and Dentofacial Orthopedics, School of Dental Medicine, University of Bern, CH-3010 Bern, Switzerland; 5 Faculty of Dentistry, Universitas Indonesia, Jakarta, 10430 Indonesia; 6 Department of Orthodontics, W.J. Kolff Institute, University of Groningen, University Medical Center Groningen, Groningen, The Netherlands

**Keywords:** Airway, CBCT, Dentofacial Orthopedics, Orthodontics, Volumetric changes

## Abstract

**Objective:**

To provide a critical overview of the effect of various orthodontic and/or dentofacial orthopedic interventions on three-dimensional volumetric changes in the upper airway.

**Materials and methods:**

Four databases were searched for clinical studies concerning 3D volumetric assessments based on CBCT before and after orthodontics interventions. The quality of the studies was assessed using the quality assessment tool of the National Heart, Lung and Blood Institute. After the use of inclusion and exclusion criteria, the pre-and post-treatment volumes were used to visualize the effect of various orthodontics interventions.

**Results:**

A total of 48 studies were included in this review and none of which were RCTs. The quality of all included studies was assessed as medium. Overall, there is a tendency for an increase in airway volumes after various orthodontic interventions, except for studies concerning extraction therapy with fixed appliances in adults, in which both increases and decreases in airway volumes have been reported.

**Conclusion:**

Orthodontic treatment by growth modification and non-extraction therapy with fixed appliances, regardless of the malocclusion, generally showed positive effects on the airway volume. Orthodontic treatment in combination with extractions does not provide an unambiguous insight. A consensus on the methodology of the airway measurement and nomenclature is urgently needed in order to gain insight into the effect of different interventions on three-dimensional airway changes.

**Clinical relevance:**

Various orthodontic treatments do not negatively influence the upper airway volume. However, extraction therapy in adults should be chosen with caution, especially in subjects belonging to a group susceptible to airway obstruction.

**Supplementary Information:**

The online version contains supplementary material available at 10.1007/s00784-023-05207-8.

## Introduction

The primary objective of orthodontic treatment is to establish an optimal dental and/or skeletal relationship in harmony with the morphology and function of the soft tissues in the oro-maxillofacial region. In addition, facilitating the development and functional demands of the airway is an important objective, especially in patients susceptible to airway obstruction or sleep apnea. Already in 1907, at the onset of orthodontics being established as a dental specialty, Angle postulated that children with a retrognathic mandible could have a smaller airway dimension. Recent studies showed that in patients with obstructive sleep apnea the underlying skeletal deformities are indeed related to a relatively restricted upper airway dimension [1–6].

Traditionally, airway dimensions were assessed using lateral cephalograms [7]. However, cephalometric measurements have severe limitations in accessing the airway, as only changes in the sagittal and vertical dimensions can be observed. Thereby neglecting the volumetric- and transversal dimensions of the airway. Moreover, 2D cephalometric and 3D volumetric measurements of the airway on CBCT [8, 9] are not a correlated. Accurate determination of the airway dimensions on a lateral cephalogram is difficult because of a large variation in 2D airway landmarks. As a better alternative, a CT, CBCT, or MRI scan could be used to assess the airway in all three dimensions. However, the costs of a CT or MRI scan are high, and the radiation dose of a multi-slice CT is much higher compared to a CBCT scan [10]. Also, in a CT scan, patients are usually in the supine position, resulting in an effect of gravity on soft tissues around the airway and therewith an error in the volume measurement on the scan will occur [11]. CBCT scans, in comparison, have much shorter image acquisition times, reducing the chance of movement of the patient during the acquisition, and providing the opportunity to perform measurements in volume, cross-sectional area, choke point, width, length, and anterior posterior dimensions of the airway. A recent systematic review concluded that airway measurements on CBCT scans have moderate to excellent reliability[12].

In the current literature, the effect of orthodontic treatment on volumetric changes in the upper airway provides multiple outcomes. Previous reviews on volumetric changes in the airway focused on one type of treatment intervention, e.g. extraction therapy with fixed appliances [13], maxillary expansion [14], and treatment of Class II malocclusion with functional appliances [15]. Due to the differences in intervention types and high heterogeneity in the definition of the airway and/or its segments, it is not possible to make relevant comparisons of the findings between different interventions or to provide a valid interpretation of the outcomes from these reviews. Moreover, no previous reviews have investigated the effect of orthodontic treatment of Class III malocclusion on the airway.

Here we aim to provide a systematic analysis of the effect of different orthodontic interventions, including transversal and sagittal growth modifications, and extraction and non-extraction therapies with fixed appliances, on 3D volumetric changes of the upper airway using a standardized nomenclature with reliable anatomical landmarks to determine the borders of the airway on CBCT scans.

## Methods

### Protocol and registration

The protocol is registered in the International Platform of Registered Systematic Review and Meta-analysis Protocols INPLASY (https://inplasy.com/) under number INPLASY202240017.

(DOI number 10.37766/inplasy2022.4.0017). The PRISMA 2020 checklist was used for reporting this systematic review [16, 17].

### Eligibility criteria

The research question was formulated by means of the Population, Intervention, Comparison, Outcome, and Study Design (PICOS) framework. The research question was: does the volume of the upper airway change after orthodontic intervention?

- P: growing subjects, adults
- I: orthodontic treatment, dentofacial orthopedics, extractions
- C: untreated subjects and/or subjects having fixed appliances treatment with non-extractions
- O: volumetric changes of the upper airway measured on CBCT scans
- S: randomized controlled trials (RCTs), controlled clinical trials, prospective cohort studies, observational studies, intervention studies

Inclusion criteria were: healthy human subjects aged 7 years and older, of any sex and with any types of orthodontic malocclusion; Subjects have had one or more of the following interventions: full orthodontic treatment with fixed appliances, or aligners with or without extraction of premolars, transversal growth modification with expansion appliances, sagittal growth modification of Class II or Class III malocclusions with functional appliances; Randomized controlled trials (RCT's), controlled clinical trials, prospective cohort studies, observational studies, intervention studies with orthodontics as intervention; Treatment group > 10 participants; CBCT acquisition with the patient positioned upright, and pre-and post-treatment 3D volumetric assessments of the airway available with clear definition or illustration of the airway.

Exclusion criteria: subjects with syndromes, cleft lip and/or palate, systemic diseases relating to orofacial growth, or OSAS and/or other airway diseases.

### Information sources and search strategy

A search was conducted in the electronic databases of PubMed, EMBASE, Web of Science, and the Cochrane Library. The 1^st^ of April 2023 was marked as the end date of the search. The search strategy for each database was as follows:

- **PubMed:**
- (‘orthodontics’[Mesh] OR orthodont*[tiab] OR dentofacial*[tiab])
- AND
- (‘respiratory System’[Mesh] OR respirat*[tiab] OR airway*[tiab] OR pharynx*[tiab] OR nasopharynx*[tiab] OR oropharynx*[tiab] OR hypopharynx*[tiab])
- **EMBASE:**
- ('orthodontics'/exp OR (orthodont* OR dentofacial*):ab,ti,kw)
- AND
- ('respiratory system'/exp OR (respirat* OR airway* OR pharynx* OR nasopharynx* OR oropharynx* OR hypopharynx*):ab,ti,kw)
- **Web of Science:**
- TS = (orthodont* OR dentofacial*)
- AND
- TS = (respirat* OR airway* OR pharynx* OR nasopharynx* OR oropharynx* OR hypopharynx*)
- **Cochrane:**
- (orthodont* OR dentofacial*)
- AND
- (respirat* OR airway* OR pharynx* OR nasopharynx* OR oropharynx* OR hypopharynx*)

All studies were retrieved with no restrictions for language or article status. Eventually, the search was updated until ^1st^ April 2023. Furthermore, manual screening of the reference lists of the studies included in the systematic review was performed. Grey literature was not searched.

### Study selection

Two authors (RS and AS), working independently, reviewed titles and abstracts (unblinded) on all the exclusion criteria. When this was insufficient the full text was screened only on exclusion criteria. The full text of the remaining articles was independently screened by the same two authors on the inclusion criteria. To be included all inclusion criteria must be met. In case of disagreement, a consensus was reached by discussion, or the third reviewer (YR) was consulted if needed. All studies were exported to an open-source reference manager software Zotero (Center for History and New Media version 6.0.19).

### Data items and data collection process

A data extraction form was developed and piloted in Covidence. Two reviewers (RS, AS) extracted the data from the included studies. Data were extracted for volumetric measurements before and after treatment intervention. If disagreement existed, it was resolved through discussion with the third reviewer (YR).

### Summary measures

Volumetric changes of the total upper airway and of its individual segments, as measured on CBCT scans were selected as the main (primary) outcome measure. Mean volumetric changes in mm^3^ were used and if available, the standard deviation (SD) from the original publication.

Comparisons of the effect on the airway of different orthodontic/orthopedic intervention categories were selected as the additional/secondary outcome.

#### Anatomical landmarks, borders, and reference planes of the airway

**Fig. 1:**
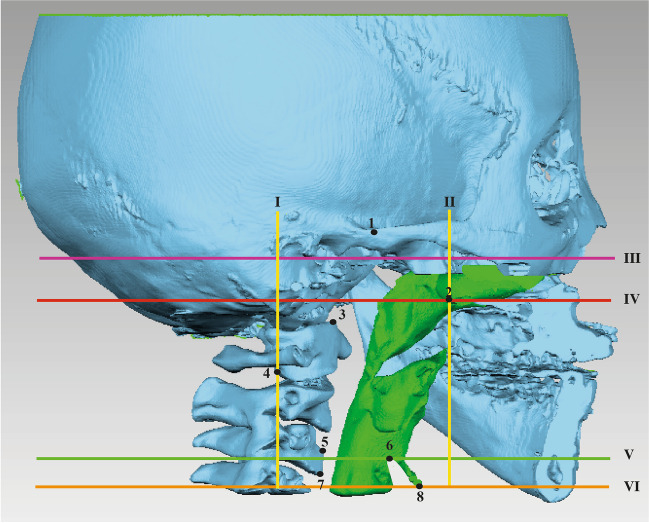
Definition of the upper airway and its segments used in this systematic review for data analysis of the included studies. The purple line indicates the most superior border of the Airway. The Red line indicates the lower border of the Nasopharynx and the upper border of the oropharynx. The green line indicates the lower border of the Oropharynx and upper border of the hypopharynx, and the orange line indicates the most inferior border of the hypopharynx. 1 = most inferior point of the floor of the sphenoid sinus, 2 = Posterior Nasal Spine, 3 = anterior superior part of C2, 4 = posterior inferior part of the C2, 5 = superior anterior part of C4, 6 = superior part of the epiglottis, 7 = anterior inferior part of C4, 8 = bottom of the epiglottis

Considering the large heterogeneity and inconsistency in the definition of the upper airway and its segments, we defined for data analysis, five cross-sectional planes (two frontal and three axial). These are based on five soft and hard tissue anatomical landmarks on the mid-sagittal plane (Fig. 1 and Table 1).

**Table 1:** Description of the upper airway and its segments used in this systematic review for data analysis of the included studies

Planes				
C2P plane	I		Frontal plane perpendicular to FH, passing through the most posterior part of the second cervical vertebra	
PNS frontal plane	II		Frontal plane perpendicular to FH, passing through PNS	
Sphenoid sinus (SS) plane	III		Axial plane parallel to FH, passing through the most inferior part of the floor of the sphenoid sinus	
PNS plane	IV		Axial plane parallel to FH, passing through PNS	
Epiglottis (E) plane	V		Axial plane parallel to FH, passing through most superior part of the epiglottis	
EF plane	VII		Plane parallel to the FH passing through the bottom of the epiglottis	
Lateral planes			MS plane: Sagittal plane perpendicular to FH, passing through the lateral surfaces of the maxillary sinus (left and right)	
Borders	Total Airway	Nasopharynx	Oropharynx	Hypopharynx
Superior	SS plane	SS plane	PNS plane	E plane
Inferior	E plane	PNS plane	E plane	EF plane
Anterior	PNS frontal plane	PNS frontal plane	PNS frontal plane	PNS frontal plane
Posterior	C2P Plane	C2P Plane	C2P plane	C2P plane
Lateral	MS Plane	MS Plane	MS plane	MS plane

#### Reference fields for the upper airway and its segments

**Fig. 2:**
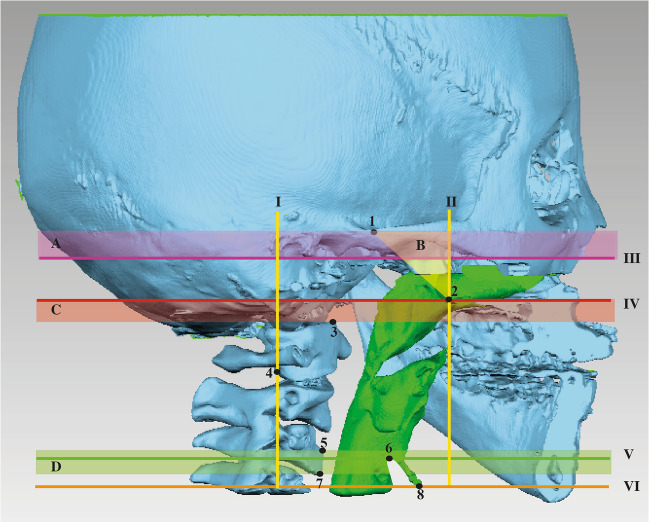
Reference fields for the upper airway and its segments. Each color block represents a ‘reference field’ that accommodates a pre-defined, limited range of variations of the respective reference plane (line in the same color). The yellow triangle indicates variations of the anterior borders accepted for data analysis in this review (**B**), the purple box indicates variations of the superior border of the nasopharynx (**A**), the red box indicates variations of the superior borders of the oropharynx (**C**), and the green box indicates variations of the inferior borders of the oropharynx (**D**)

Data retrieved from the original studies were standardized following a previously published protocol, based on the concept of ‘reference fields’ that accommodates a pre-defined, limited range of variations in the reference plane [18]. Briefly, the anatomical landmarks and reference planes used in the original studies were compared to the proposed reference fields that are illustrated in Fig. 2.

#### Volumetric data inclusion and interpretation using the reference fields

The following protocol was applied on pre-, and post-treatment volumetric data extracted from the included studies using the reference fields described above.

1. Data inclusion without additional validation: original data were included directly when the definition of the airway and its segments concurs with the proposed reference planes (Table 1, Fig. 1).
2. Data inclusion after additional validation (in *italics* in Table 3): original data were included when the definition of the airway and its segments falls within the proposed reference fields (Fig. 2).
3. Data exclusion: original data were excluded when the definition of the airway and its segments falls outside the proposed reference fields (Fig. 2).

In the case of multiple post-treatment follow-ups, the longest follow-up results were used.

### Risk of bias in individual studies

The quality of the included studies was assessed according to the quality assessment tool of the National Heart, Lung, and Blood Institute (https://www.nhlbi.nih.gov/health-topics/study-quality-assessment-tools). Depending on the type of study, the quality assessment tool for “Case-control Studies” or, if applicable, for “Before-After (Pre-Post) Studies with no control group” was used. Rating of a study was done according to a questionnaire of twelve questions, answered by ‘yes’ or ‘no’, whereas ‘yes’ scores one point and ‘no’ scores no point. A maximum of 12 points could be obtained. A score of 1–4 qualified as poor, 5–9 as fair, and 10–12 as good. Two reviewers performed the rating independently (RS, AS).

Disagreements were discussed and solved with a third author (YR).

### Additional analysis

A bar graph was generated to visualize the relative changes in the airway and its segments resulting from different types of orthodontic interventions.

### Planned methods of analysis

First, heterogeneity between the studies was assessed based on population, age, treatment, and follow-up period. Due to a large heterogeneity between studies, a quantitative analysis was not possible, and a descriptive synthesis was conducted.

## Results

### Study selection (Fig. 3)

**Fig. 3:**
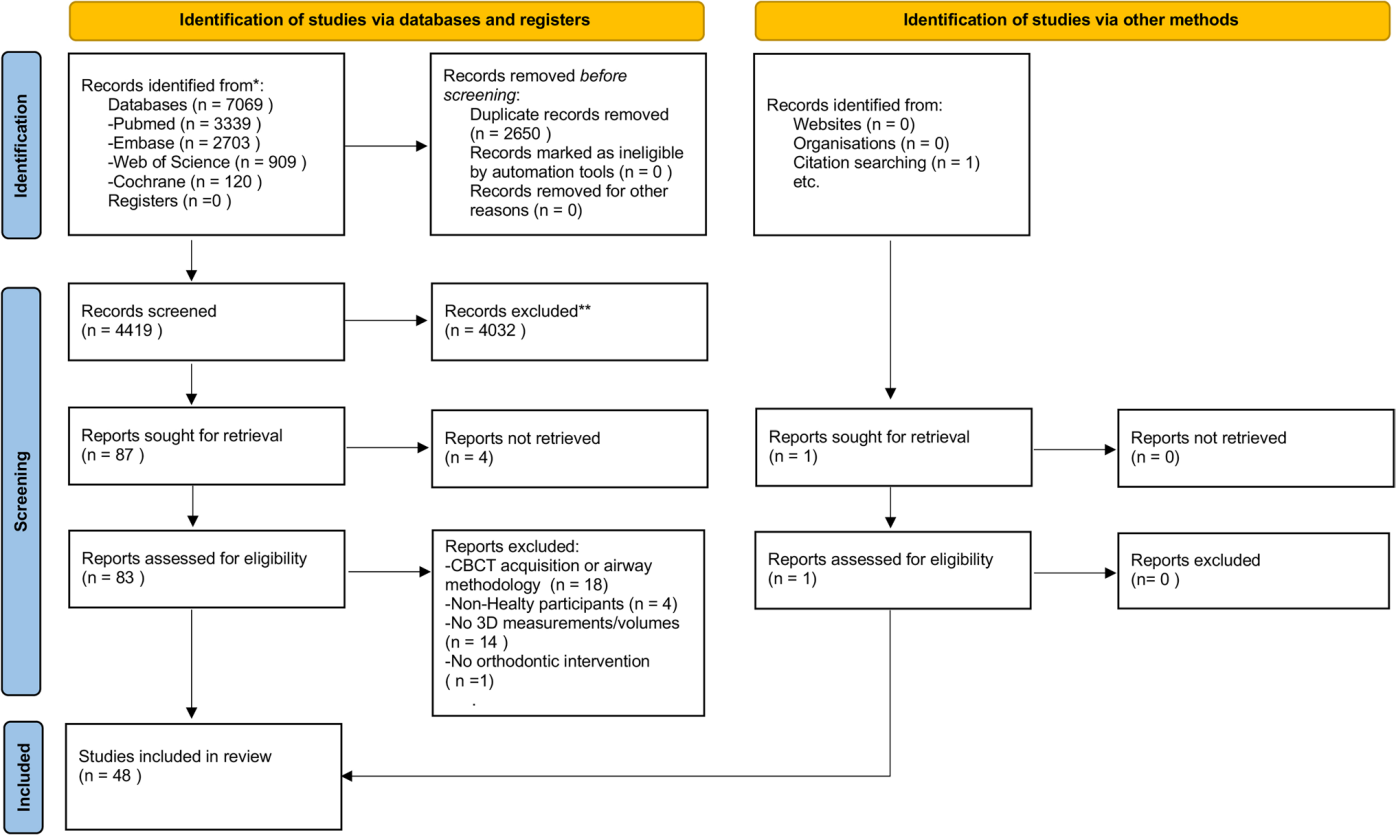
PRISMA 2020 flow chart representing the study selection process

A total of 7069 articles were retrieved after the first search with one additional hit after a hand search or from citations. Figure 3 illustrates the PRISMA 2020 Flow Diagram and a detailed overview of the selection process. After the removal of the duplicates, 4419 articles remained for further screening of titles and abstracts. A total of 88 articles were eligible for the full-text assessment of the inclusion criteria. Out of the 88 articles, 4 full texts were not retrievable. Of these, 35 studies were excluded due to predetermined exclusion criteria. Finally, after the additional hand search, 50 studies met the inclusion criteria for this systematic review.

### Study characteristics (Table 2)

**Table 2:** Characteristics of the included studies. In study design, R = retrospective, P = prospective, M = multi-center, S = single-center study; / indicates no data reported; follow-up: mean time in months between the first and latest available CBCT scans; quality of the study according to the quality assessment tool for “Case-Control studies” or “Before-After (pre-post) studies with no control group” of the National Heart, Lung and Blood Institute. In column 1 an a or b is added to the reference number when a study is mentioned multiple times in the table; [19]a: Bone-born expansion; [19]b: Tooth-born expansion; [20]a: Slow Maxillary Expansion with a Leaf expander; [20]b: Rapid Maxillary Expansion with a Hyrax; [21]a: Tooth-born expansion; [21]b: Bone-born expansion; [22]a: 0.8 mm expansion per day; [22]b: 0.5 mm expansion per day; [23]a: Rapid Maxillary Expansion [23]b: Rapid Maxillary Expansion and constriction; [24]a: Hybrid Hyrax (Tooth- and Bone born) expansion and protraction; [24]b; Tooth-born expansion and protraction; [25]a: Twin block treatment [25]b: Fixed functional appliance, Forsus fatigue resistance device; [26]a: intermaxillary elastics; [26]b: Fixed functional appliance Forsus fatigue resistance device. [27]a: normal vertical growth [27]b: hyper-divergent vertical growth

Author	Year	Study design	Multi or single center	Treatment subjects			Control group		Angle Class	Follow-up Mean ± SD (months)	Quality
				N and Sex	Age	Sample size calculation	N and Sex	Age (mean ± SD)			
1.1 Maxillary transversal growth modification (*N* = 27)											
Abdalla et al. [28]	2019	R	S	*N* = 26 12 M 14F	12.3 ± 2.3	Yes, *N* = 21	*N* = 26 12 M 14F	12.3 ± 2.3	/	24 ± 11	8
Almuzian et al. [29]	2018	R	?	*N* = 17 8 M 9F	M = 12.4 F = 12.8	Yes, *N* = 17	No control group	/	/	1	10
Chang et al. [30]	2013	P	S	*N* = 14 5 M 9F	12.9 Range 9.7–16	No	No control group	/	/	**≈**5	9
El et al. [31]	2014	R	S	*N* = 35 20 M 15F	M = 14 ± 1.5 F = 14.0 ± 1.5 Total = 14.0 ± 1.5	No	*N* = 35 20 M 15F	M = 14.0 ± 1.8 F = 14.3 ± 0.8 Total = 14.1 ± 1.4	I	26 ± 4	7
Iwasaki et al. [32]	2013	R	S	*N* = 28 13 M 15F	10.0 ± 1.2	No	*N* = 20 8 M 12F	9.7 ± 1.0	II	6 ± 1.0	7
Kavand et al. [19]a	2019	R	S	*N* = 18 8 M 10F	14.4 ± 1.3	Yes, *N* = 20	*N* = 18 6 M 12F	14.7 ± 1.4	/	**≈**3	8
Kavand et al. [19]b	2019	R	S	*N* = 18 6 M 12F	14.7 ± 1.4	Yes, *N* = 20	*N* = 18 8 M 10F	14.4 ± 1.3	/	**≈**3	8
Kim SY et al. [33]	2018	R	S	*N* = 14 4 M 10F	22.7 ± 3.3	No	No control group	/	/	**≈**14	8
Zeng et al. [34]	2013	P	S	*N* = 16 10 M 6F	12.7 ± 1.7	No	No control group	/	/	3	9
Yilmaz et al. [35]	2015	R	S	*N* = 20 10 M 10F	9. 8	No	No control group	/	III	≈2	7
Lanteri et al. [20]a	2020	R	S	*N* = 22 9 M 13F	M = 7.9 ± 0.4 F = 8.1 ± 0.7	Yes, *N* = 18	*N* = 22 11 M 11F	M = 8.4 ± 0.9 F = 8.1 ± 0.7	/	11.4	8
Lanteri et al. [20]b	2020	R	S	*N* = 22 11 M 11F	M = 8.4 ± 0.9 F = 8.1 ± 0.7	Yes, *N* = 18	*N* = 22 9 M 13F	M = 7.9 ± 0.4 F = 8.1 ± 0.7	/	11.4	8
Yi et al. [36]	2020	R	S	*N* = 13 3 M 10F	19.6 ± 5.3	No	No control group	/	/	± 3	5
Gianoni et al. [37]a	2020	P	S	*N* = 29 12 M 17F	13.2	Yes, *N* = 12	*N* = 31 11 M 20F	13.9	/	22	9
Gianoni et al. [37]b	2020	P	S	*N* = 31 11 M 20F	13.9	Yes, *N* = 12	*N* = 29 12 M 17F	13.2	/	22	9
Fastuca et al. [38]	2015	P	S	*N* = 15 4 M 11F	7.5 ± 0.3	Yes, *N* = 10	No control group	/	/	12	9
Mehta et al. [21]a	2021	R	S	*N* = 20	13.7 ± 1.7	Yes, *N* = 19	*N* = 19	13.3 ± 1.5	/	33.6	9
Mehta et al. [21]b	2021	R	S	*N* = 21	13.9 ± 1.1	Yes, *N* = 19	*N* = 19	13.3 ± 1.5	/	34.8	9
Caprioglio et al. [39]	2014	P	S	*N* = 14	7.1 ± 0.6	Yes, *N* = 10	No control group	/	/	12	7
Niu et al. [40]	2021	R	M	*N* = 39	10.4 ± 1.7	No	*N* = 29	11.1 ± 1.5	/	22.6 ± 10.3	8
Lofti et al. [22]a	2018	R	S	N20 12F 8 M	12.3 ± 1.9	No	*N* = 20 10F 10 M	13.8 ± 1.3	/	3	9
Lofti et al. [22]b	2018	R	S	*N* = 20 10F 10 M	13.8 ± 1.3	No	N20 12F 8 M	12.3 ± 1.9	/	3	9
Aljawad et al. [41]	2021	R	S	*N* = 17 14F 3 M	12.6 ± 1.8	Yes, *N* = 17	*N* = 17 13F 4 M	12.3 ± 1.5	I: 9 II: 2 III: 6	10.5 ± 5.3	7
Ribeiro et al. [42]	2011	R	S	*N* = 15 8F 7 M	7.5	No	No control group	/	/	4	6
DiCosimo et al. [43]	2021	R	S	*N* = 28 17F 11 M	9.9 ± 2.4	No	*N* = 20 11F 9 M	10.4 ± 1.6	/	20.6 ± 2.1	7
Chang et al. [23]a	2017	R	S	*N* = 18 11F 7 M	9.8 ± 1.3	No	*N* = 18 11F 7 M	9.8 ± 1.3	III	± 3	9
Chang et al. [23]b	2017	R	S	*N* = 16 10F 6 M	10.3 ± 1.6	No	*N* = 16 10F 6 M	10.3 ± 1.6	III	± 3	9
1.2 Sagittal growth modification of Angle Class III malocclusion (*N* = 6)											
Chen et al. [44]	2015	P	S	*N* = 30 16 M 14F	9.6 ± 0.2	No	*N* = 30 16 M 14F	10.4 ± 0.4	III	10	9
Pamporakis et al. [45]	2014	R	S	*N* = 22	10 y	No	No control group	/	III	/	7
Liu et al. [46]	2021	P	S	*N* = 20 9 M 11F	8–10 y	No	No control group	/	III	12	10
Miranda et al. [24]a	2022	P	S	*N* = 20 12 M 8F	10.8 ± 1	Yes, *N* = 9	*N* = 15 9 M 6F	11.5 ± 1.2	III	11 ± 4	10
Miranda et al. [24]b	2022	P	S	*N* = 15 9 M 6F	11.5 ± 1.2	Yes, *N* = 9	*N* = 20 12 M 8F	10.8 ± 1	III	11 ± 4	10
Nguyen et al. [47]	2015	R	S	*N* = 28 14F 14F	11.9 ± 1.2	No	*N* = 29 16F 12 M	12.4 ± 1.2	III	14	7
1.3 Sagittal growth modification of Angle Class II malocclusion (*N* = 13)											
Iwasaki et al. [48]	2014	R	S	*N* = 24 11 M 13F	11.6 ± 0.9	No	*N* = 20 9 M 11F	11.5 ± 0.7	II	42	9
Li et al. [49]	2014	R	S	*N* = 30 13 M 17F	11.6 ± 0.9	No	*N* = 30 13 M 17F	11.7 ± 0.9	II	14 ± 1	9
Temani et al. [50]	2016	R	S	*N* = 30	10–17	No	No control group	/	II	/	8
Erbas et al. [51]	2014	R	S	*N* = 25 11 M 14F	11.1 ± 1.1 9.1–12.5	No	No control group	/	II	6	8
Alhammadi et al. [25]a	2019	R	S	*N* = 23 23F	11.9 ± 1.9	Yes, *N* = 20	*N* = 18 18F	11.3 ± 1.2	II	/	8
Alhammadi et al. [25]b	2019	R	S	*N* = 21 23F	13.5 ± 1.1	Yes, *N* = 20	*N* = 18 18F	11.3 ± 1.2	II	/	8
Oliveira et al. [52]	2020	R	S	*N* = 24 15 M 9F	13.8 ± 1.2	Yes, *N* = 18	*N* = 18 10 M 8F	/	II	9 ± 1	8
Abdalla et al. [53]	2020	R	S	*N* = 73 36 M 37F	12.0 ± 1.5	No	*N* = 73 36 M 37F	12.0 ± 1.5	II	23 ± 11	7
Chou et al. [54]	2021	R	S	*N* = 20 8 M 12F	12.9 ± 1.0	No	*N* = 20 11 M 9F	19.3 ± 1.6	II	13.9 ± 2.3	8
Xiao et al. [55]	2020	R	S	*N* = 37 22F 15 M	24.6 ± 5.1	No	No control group	/	II	± 18	6
Thereza-Bussolaro et al. [26]a	2019	R	M	*N* = 15 9F 6 M	15.2	Post-hoc power analysis; appropriate power	*N* = 14 9F 5 M	12.4	II	± 13	7
Thereza-Bussolaro et al. [26]b	2019	R	M	*N* = 14 9F 5 M	12.4	Post-hoc power analysis; appropriate power	*N* = 15 9F 6 M	15.2	II	± 21	7
Abdalla et al. [56]	2022	R	S	*N* = 42	12.0 ± 1.5	Yes, *N* = 18	*N* = 63 31 M 32F	12.0 ± 1.5	II	23	9
2 Non-extraction therapy with fixed appliances or aligners (*N* = 14)											
Abdalla et al. [28]	2019	R	S	*N* = 26 12 M 14F	12.3 ± 2.3	Yes, *N* = 21	*N* = 26 12 M 14F	12.3 ± 2.3	I	24 ± 11	8
El et al. [31]	2014	R	S	*N* = 35 20 M 15F	M = 14.0 ± 1.8 F = 14.3 ± 0.8 Total = 14.1 ± 1.4	No	*N* = 35 20 M 15F	M = 14 ± 1.5 F = 14.0 ± 1.5 Total = 14.0 ± 1.5	I	26 ± 4	7
Iwasaki et al. [32]	2013	R	S	*N* = 28 13 M 15F	9.7 ± 1.0	No	*N* = 28 13 M 15F	10.0 ± 1.2	II	6 ± 1	7
Iwasaki et al. [48]	2014	R	S	*N* = 20 9 M 11F	11.5 ± 0.7	No	*N* = 24 11 M 13F	11.6 ± 0.9	I	40	9
Abdalla et al. [53]	2020	R	S	*N* = 73 36 M 37F	12.0 ± 1.5	No	*N* = 73 36 M 37F	12.0 ± 1.5	I	23 ± 10	7
Abdalla et al. [56]	2022	R	S	*N* = 63 31 M 32F	12.0 ± 1.5	Yes, *N* = 18	*N* = 42	12.0 ± 1.5	I	23	
Park et al. [57]	2018	R	S	*N* = 17	21.5	No	*N* = 16	22.9	II	/	8
Pliska et al. [58]	2016	R	S	*N* = 48 17 M 31F	31.9 ± 12.0	No	*N* = 26 8 M	27.4 ± 9.7	I and II	19 ± 5	8
Valiathan et al. [59]	2010	R	S	*N* = 20 10 M 10F	M13.8 ± 1.2 F13.5 ± 1.6	No	*N* = 20 10 M 16F	M13.8 ± 1.3 F13.5 ± 1.6	I	25 ± 4	8
Stefanovic et al. [60]	2013	R	S	*N* = 31 15 M 16F	12.9 ± 0.7	No	*N* = 31 15 M 16F	13.0 ± 1.2	I, II and III	28 ± 4	5
Joy et al. [61]	2020	R	S	*N* = 42 22 M 20F	26.0 ± 8	No	*N* = 41 20 M 21F	26.1 ± 7.1	I, II and III	28 ± 11	7
Chen et al. [62]	2017	R	S	*N* = 25 15F 10 M	12.4 ± 1.5	No	*N* = 25 15F 10 M	12.2 ± 1.2	I	/	8
Guo et al. [63]	2022	R	S	*N* = 40	26.4 ± 4.9	No	*N* = 120	25.1	I/II	24 ± 4	7
3 Extraction therapy with fixed appliances or aligners (*N* = 11)											
Park et al. [57]	2018	R	S	*N* = 16	22.9	No	*N* = 17	21.5	II	/	8
Pliska et al. [58]	2016	R	S	*N* = 26 8 M	27.4 ± 9.7	No	*N* = 48 17 M 31F	23.5 ± 4.5	I and II	24 ± 5	8
Valiathan et al. [59]	2010	R	S	*N* = 20 10 M 10F	M13.8 ± 1.3 F13.5 ± 1.6	No	*N* = 20 10 M 10F	M13.8 ± 1.2 F13.5 ± 1.6	I	31 ± 4	8
Stefanovic et al. [60]	2013	R	S	*N* = 31 15 M 16F	13.0 ± 1.2	No	*N* = 31 15 M 16F	12.9 ± 0.7	I, II and III	33 ± 5	5
Joy et al. [61]	2020	R	S	*N* = 41 20 M 21F	26.1 ± 7.1	No	*N* = 42 22 M 20F	26.0 ± 8	I, II and III	42 ± 19	7
Chen et al. [62]	2017	R	S	*N* = 25 15F 10 M	12.2 ± 1.2	No	*N* = 25 15F 10 M	12.4 ± 1.5	I	/	8
Guo et al. [63]	2022	R	S	*N* = 120	25.1	No	*N* = 40	26.4 ± 4.9	I/II	32	7
Zhang et al. [64]	2015	R	S	*N* = 18 5 M 13F	24.1 ± 3.8 18–33	No	*N* = 18	/	II	30	8
Shi et al. [65]	2021	R	S	*N* = 18 7 M 11F	21.2 ± 2.9	No, only post-hoc power analysis	No control group	/	II	/	9
Ning et al. [27]a	2022	R	S	*N* = 29	20–35	No	*N* = 28	20–35	II	/	9
Ning et al. [27]b	2022	R	S	*N* = 28	20–35	No	*N* = 29	20–35	II	/	9

In Table 2 the characteristics of the total of 48 included studies are presented. From these 48 studies, 71 treatment groups (N) were identified and divided into the following three intervention categories:

1. Non-extraction growth modification (*N* = 46);
  1.1 Maxillary transversal growth modification (*N* = 27)
  1.2 Sagittal growth modification of Angle Class III malocclusion (*N* = 6)
  1.3 Sagittal growth modification of Angle Class II malocclusion (*N* = 13)
2. Non-extraction therapy with fixed appliances or aligners without prior dentofacial orthopedic therapy (*N* = 14).
3. Extraction therapy with fixed appliances or aligners without prior dentofacial orthopedic therapy (*N* = 11).

The studies on growth modification involved only growing patients (1.1, 1.2, 1.3), while those using fixed appliances or aligners involved both growing and adult subjects (2 and 3). Follow-up in the studies varied from 1 month up to 42 months, with 24 months being the most frequent follow-up.

### Risk of bias within studies (Table 2)

In 48 included studies, only 16 reported a power-analysis (or post-hoc analysis) to determine the minimal number of subjects needed. No randomized controlled trials could be identified. Except for one unknown [29] and two multi-center studies [26, 40], all included studies were single-center based. Eight studies had a prospective and 40 a retrospective study design. Four studies had an untreated control group with both pre-and post-treatment CBCT scans [21, 25, 43, 52]. Three studies included an untreated control group, with only post-treatment CBCT scans available [44, 49, 54]. In five studies on growth modification, age-matched subjects treated with ‘non-extraction fixed appliances’ served as a control [28, 31, 32, 48, 53]. In six other studies, subjects with ‘extraction fixed appliances’ were compared to subjects with ‘non-extraction fixed appliances’ [56–62].

Three studies were rated as ‘good’ (score 10), and the other included studies were qualified as ‘medium risk of bias’. Forty-four studies scored between 5 to 9, indicating ‘fair quality’. No studies scored under 5 points (poor quality).

### Main outcomes

#### Airway volumetric changes in relation to different interventions

Airway volumetric changes in mm^3^ after different types of interventions are presented in Table 3. Among the three airway segments, oropharynx volumes were reported in all studies except five [20, 33, 38, 39, 42] on maxillary transversal expansion, one on Class III growth modifications [45], one on fixed appliances treatment [61] and one on fixed appliances with extractions [64]. Nasopharynx volumes were reported in more than half of the studies on maxillary transversal expansion but in less than half of the other treatment groups. Only five studies reported the volumes on the hypopharynx airway [22, 23, 28, 46, 49].

**Table 3:** Volumetric changes after treatment in mm^3^ and in %. All volumetric changes are mean values in mm^3^ unless otherwise indicated. Next to the difference between pre-and post-treatment volumes in mm^3^, a relative change in percentage is presented

Volumetric change after treatment in mm^3^ and in %								
Ref	Total Airway		Nasopharynx		Oropharynx		Hypopharynx	
	Mm^3^	%	Mm^3^	%	Mm^3^	%	Mm^3^	%
1.1 Maxillary transversal growth modification (*N* = 27)								
[28]	-	-	-	*-*	*4587* *	35,6%	-	-
[29]	*19*	0,0%	365 *	*13,4%*	*-346*	-9,0%	-	-
[30]	-	-	-	*-*	*1735* ± *5971*	15,5%	-	-
[31]	-	-	-	*-*	*1273* ± 1676 **	16,8%	-	-
[32]	-	-	-	-	3015 ± 1298 *#	47,3%	-	-
[19]a	*1371*	8,8%	820 ± 275 *	21,8%	551 ± 620	0,5%	-	-
[19]b	*992*	6,8%	708 ± 159 *	20,0%	284 ± 386	2,6%	-	-
[33]	*942* ± *821* *	6,6%	-	-	-	-	-	-
[20]a	*-*	-	*1743* ± *680*	42,2%	-	-	-	-
[20]b	*-*	-	*1684* ± *810*	47,6%	-	-	-	-
[36]	*-644* ± *6133*	*-2,4%*	*502* ± 975 *	*8,5%*	*-1085* ± *5477*	5,2%	-	-
[37]a	*-*	*-*	*-*	*-*	2230*	18,1%	-	-
[37]b	*-*	*-*	*-*	*-*	*1000*	7,5%	-	-
[38]	*3449*	13,3%	-	-	-	-	-	-
[21]a	*3810*	*23,3%*	1359 **	*44,3%*	*2451*	19,0%	-	-
[21]b	*2271*	*14,0%*	856 **	*29,0%*	*1415*	11,1%	-	-
[39]	*2671*	*28,5%*	*-*	*-*	*-*	-	-	-
[40]	*2085*	*26,0%*	454 **	*34,9%*	*1631*	2–4,3%	-	-
[22]a	*551*	*3,9%*	*456* ± 803 **	*12,4%*	*95* ± *595*	*0,9%*	*-21* ± *133*	*-0,9%*
[22]b	*175*	*1,2%*	*103* ± *352*	*0,4%*	*72* ± *958*	*0,6%*	*-3* ± *141*	*-0,2%*
[41]	*2518*	*19,9%*	*658* ± *1028*	*21,1%*	*1859*	19,4%	-	-
[42]	*1119*	*12,3%*	*-*	*-*	*-*	-	-	-
[43]	*3348*	*32,0%*	*1000* ± 918 **	*43,9%*	*2349* ± 2520 **	33,8%	-	-
[23]a	*1844*	*14,2%*	*668* ± 877 **	*24,3%*	*1174* ± *4314*	*11,5%*	*116* ± *517*	*5,7%*
[23]b	*707*	*4,7%*	*607* ± 753 **	*21,4%*	*100* ± *2852*	*0,8%*	*153* ± *434*	*6,7%*
1.2 Sagittal growth modification of Angle Class III malocclusion (*N* = 6)								
[44]	*1879*	16,7%	525 *	*13,5%*	*1357* *	18,4%	-139	-6,1%
[45]	*407*	4,5%	-	-	-	-	-	-
[46]	4194 **	27,5%	842 **	*22,8%*	*2553*	28,2%	797	32,0%
[24]a	-	-	-	-	2873*	*23,5%*	-	-
[24]b	-	-	-	-	*2561*	*20,7%*	-	-
[47]	-	-	-	-	1499 *	11,9%	-	-
1.3 Sagittal growth modification of Angle Class II malocclusion (*N* = 13)								
[48]	-	-	-	-	*9187*	219%	-	-
[49]	*2303*	27,3%	576	18,7%	1727 #	32,3%	500 #	28,3%
[50]	-	-	-	-	1601 *	20,4%	-	-
[51]	-	-	-	-	1744 (median) *	35,7%	-	-
[25]a	*3776*	22,8%	507 *#	*14,0%*	3270 *#	25,3%	-	-
[25]b	*-70*	-0,4%	-87	*2,0%*	*16*	0,1%	-	-
[52]	*5360*	23,2%	980 ± 2330	*11,8%*	*4380* ± *6346* *#	29,7%	-	-
[53]	-	-	-	-	5659 *#	54,0%	-	-
[54]	-	-	-	-	*1600*	14,7%	-	-
[55]	*5250*	*17,0%*	*161*	*2,2%*	*5092*	21,5%	-	-
[26]a	-	-	-	-	*2354* ± 4059 *	32,9%	-	-
[26]b	-	-	-	-	*2192* ± *4452*	28,7%	-	-
[56]	-	-	-	-	7759 #	65,0%	-	-
2 Non-extraction therapy with fixed appliances or aligners (*N* = 14)								
[28]	-	-	-	*-*	3578 *	29,3%	-	-
[31]	-	-	-	*-*	*1448* ± 2464 *	18,0%	-	-
[32]	-	-	-	-	1226 ± 1783 *#	18,9%	-	-
[48]	-	-	-	-	5134	54,5%	-	-
[53]	-	-	-	-	1473	12,0%	-	-
[56]	-	*-*	-	-	-920 ± 4114	-5,7%	-	-
[57]	-1704 ± 5446	*-*	*37* ± *1140*	-	-*1509*	-9,6%	-	-
[58]	-	*-*	-	-	*1701* ± *3678*	14,2%	-	-
[59]	-	*-*	-	-	1105 *	18,3%	-	-
[60]	-	*-*	*170*	3,6%	-	-	-	-
[61]	1620	*3%*	*45* ± *314*	*2%*	*-1665*	4,0%	-	-
[62]	-	-	-	-	2600 (median) *	37,7%	-	-
[63]	-	-	-	-	176	1,1%	-	-
3 Extraction therapy with fixed appliances or aligners (*N* = 11)								
[57]	-		-	-	-530 ± 4080	-4,1%	-	-
[58]	-1366 ± 4061	-6,8%	*-136* ± *1379*	-2,3%	*-826*	5,8%	-	-
[59]	-	-	-	*-*	*1083* ± *2504*	8,5%	-	-
[60]	-	-	-	-	1669 *	33,0%	-	
[61]	*-*	*-*	*-50*	*-1%*	-	-	-	-
[62]	*292*	*-10%*	*83* ± *414*	*1%*	*375*	-15%	-	
[63]	-	-	-	-	1658*	10,3%	-	-
[64]	*-1249 (median)*	*-4,9%*	*-25 (median)*	*-0,5%*	*-961* *(median)*	*-5,3%*	-	-
[65]	-	-	-	*-*	*1168*	6,3%	-	-
[27]a	*431*	*4,4%*	*58* ± *19*	*1,3%*	*373*	*5,8%*	*513* ± *26*	6,7%*#
[27]b	*170*	*1,1%*	*-22* ± *9*	*-0,5%*	*192*	*1,9%*	*91* ± *31*	*1,1%*

* Indicates a significant(*P* < 0.05) increase or decrease compared to the pre-treatment measurement; ** Indicates a significant(*P* < 0.001) increase or decrease compared to the pre-treatment measurement; # Indicates a significant difference compared to the control group, - indicates data not available from the original studies; *Italics* indicates data inclusion after validation by the protocol

An overall increase in the airway volume was shown in studies with growth modification and fixed appliances treatment without extraction, regardless of the pre-treatment malocclusion (Table 3 Sections 1.1, 1.2, 1.3, and “Methods”).

Results for fixed appliances therapy with extraction were less consistent, with both increase and decrease of volumes in the airway being reported, though the change was significant only in one study (*p* < 0.05) [60]. This inconsistency can be related to the age of the study subjects, as a decrease in the volume of the airway was observed only in adult patients[26, 55, 58, 60] while an increase was observed mostly in growing adolescents [59, 60].

### Additional outcomes

In Supplementary files 1 to 5 bar graphs are presented to illustrate the percentages of post-treatment volumetric changes in relation to the respective pre-treatment level. The study of Iwasaki et al. reported an exceeding post-treatment volumetric increase of 219%, attributed to a very long follow-up (42 months), and was therefore excluded from the bar [48]. Patterns can be recognized for different treatment modalities. Volumes of the airway in studies with dentofacial-orthopedic growth modification showed almost all increases, up to 60% of the pre-treatment levels, regardless of the power of the study or the type of interventions. The increases were observed most frequently in the oropharynx (Supp. 1, 2 and 3). Treatment with fixed appliances showed distinguishable features in the oropharynx airway between extraction and non-extraction therapies. An overall increase of the volume was observed, up to 55% of the pre-treatment level after non-extraction therapy (Supp. 4). Extraction therapy, on the other hand, resulted in changes in both positive and negative directions, though to a lesser degree compared with non-extraction therapy (Supp. 5).

## Discussion

### Summary of evidence

Orthodontic and dentofacial orthopedic treatment modifies the position of the skeletal, dental, and soft tissues within the maxillofacial complex. Therewith the soft tissues surrounding the upper airway may adapt to a new position, resulting in volumetric changes in the airway. The present review included all eligible studies on 3D volumetric changes in the upper airway after orthodontic and/or dentofacial orthopedic interventions. A meta-analysis could not be performed due to the high level of heterogeneity in the volumetric data, resulting from large variations of the defined anatomical borders of the airway.

Results from the present review did not show any evidence of a negative impact of orthodontic interventions on airway volumes, during the observation periods. The only exception might be extraction therapy (of premolars), in which a tendency of volumetric decrease in the airway was observed in adult subjects [57, 58]. However, changes in the airway were small and statistically not significant and amounted to a maximum of—8% of the original values. Orthodontic extraction therapy is often related to the shortening of the anterior-posterior arch length and retraction of the anterior teeth. These changes may lead to the backward movement of the tongue that compresses the soft palate and narrows the oropharynx airway. However, evidence is lacking to support such a causal effect. Growing subjects may accommodate broader indications for extraction therapy, without normal growth of the airway volume being impeded during the treatment period. In comparison, studies on non-extraction therapy almost all showed a volumetric increase in the airway up to 55% of the pre-treatment level, with the largest changes seen in subjects between 9 to 12.0 years of age [28, 32].

Among the three types of growth modification therapy, the most notable change was in patients treated with maxillary expansion. In which the volumes increased in all three airway segments. In this group, the subjects were relatively young, with a range of the average ages between 7.9 to 14.7 years, except for one non-controlled study with a small sample (*N* = 13) of young adults aged 19.6 years and a follow-up of only 3 months, with a quality score of 5 [36]. An average of 13% volumetric increase was found in the airway across all included studies on maxillary expansion. This appears comparable with an average of 10% in studies on surgically assisted maxillary expansion in adults reported in a previous review [18].

In 7 out of 13 studies on growth modifications in subjects with Class II malocclusion, the post-treatment airway volumes were significantly higher than the pre-treatment level and/or the age-matched controls especially in the oropharynx. Demonstrating an additional gain from the intervention. These results are in line with a recent review, reporting weak evidence for a volumetric increase in the upper airway based on 5 studies on treatment with functional appliances in patients with Class II malocclusion [15].

Growth modifications in subjects with a Class III malocclusion showed a volumetric increase in different airway segments. All studies in this category had a reasonable quality, although two studies had no control group which means the effects of normal growth and therapy cannot be separated. In all included studies, except for the study of Liu et al. [46],a protraction force was applied to the maxilla to enhance the forward and downward growth of the maxilla. Out of 6 study groups, five demonstrated a significant increase in the volume in at least one airway segment. An average of 18% volumetric increase in the airway across all included studies in this category of intervention, is higher than that of 14% in patients undergoing a single jaw Le-Fort I advancement reported in a previous review [18], which may be attributed to a combined effect of favorable treatment reactions and normal growth in the airway.

Though some patterns could be recognized in the outcome from the present review, one has to bear in mind that volumetric changes in the upper airway are influenced by multiple factors, such as initial indications (crowding or retraction) for extraction [13], retraction of the upper- and lower incisors [57, 64] and dental alignment of crowding [60]. It is, therefore, not possible to draw a firm conclusion concerning the effect of one specific type of intervention.

### Limitations

One limitation of the current review is the wide range of follow-up lengths between the included studies. Obviously, studies with longer follow-up periods will cover a greater span of normal growth, which may result in both larger absolute volumetric measurements and relative percentual changes. Another limitation is that no randomized controlled trials could be included, even though the quality of all included studies was assessed as medium. Additionally, the absence of an untreated control group in many of the included studies is a matter of discussion, as it makes it challenging to distinguish the genuine treatment effect from normal growth.

## Conclusions and Recommendations for future research

Taking into account the acknowledged limitations, the present review concludes that orthodontic treatment, regardless of the type of intervention, malocclusion, or patient age, did not yield evidence for changes in upper airway volume whether positive or negative.

A joint endeavor in the dental community to establish a consensus on airway measurement methodology and terminology, including the various segments, will greatly enhance the quality and comparability of studies on volumetric changes in the airway. Future studies may focus on extraction therapy in adults, particularly those susceptible to airway obstruction, in order to identify potential risk factors that impede airway growth. Other clinically relevant parameters such as the average cross-sectional surface areas and choke points (minimal cross-sectional areas) in airway evaluation, in addition to volumetric measurements in cubic millimeters, may also be considered.

### Supplementary Information

Below is the link to the electronic supplementary material.

Five bar graphs of the relative changes in the airway after different orthodontic interventions.

Supplementary file1 (JPG 1501 KB)

Supplementary file2 (JPG 1221 KB)

Supplementary file3 (JPG 1327 KB)

Supplementary file4 (JPG 1280 KB)

Supplementary file5 (JPG 1254 KB)
