# Supplementary figures and images for: Volumetric changes in the upper airway on CBCT after dentofacial orthopedic interventions - a systematic review
Source: Clin Oral Investig. 2023 Sep 19;27(10):5737–54. doi: 10.1007/s00784-023-05207-8 (PMC10560168; doi:10.1007/s00784-023-05207-8)

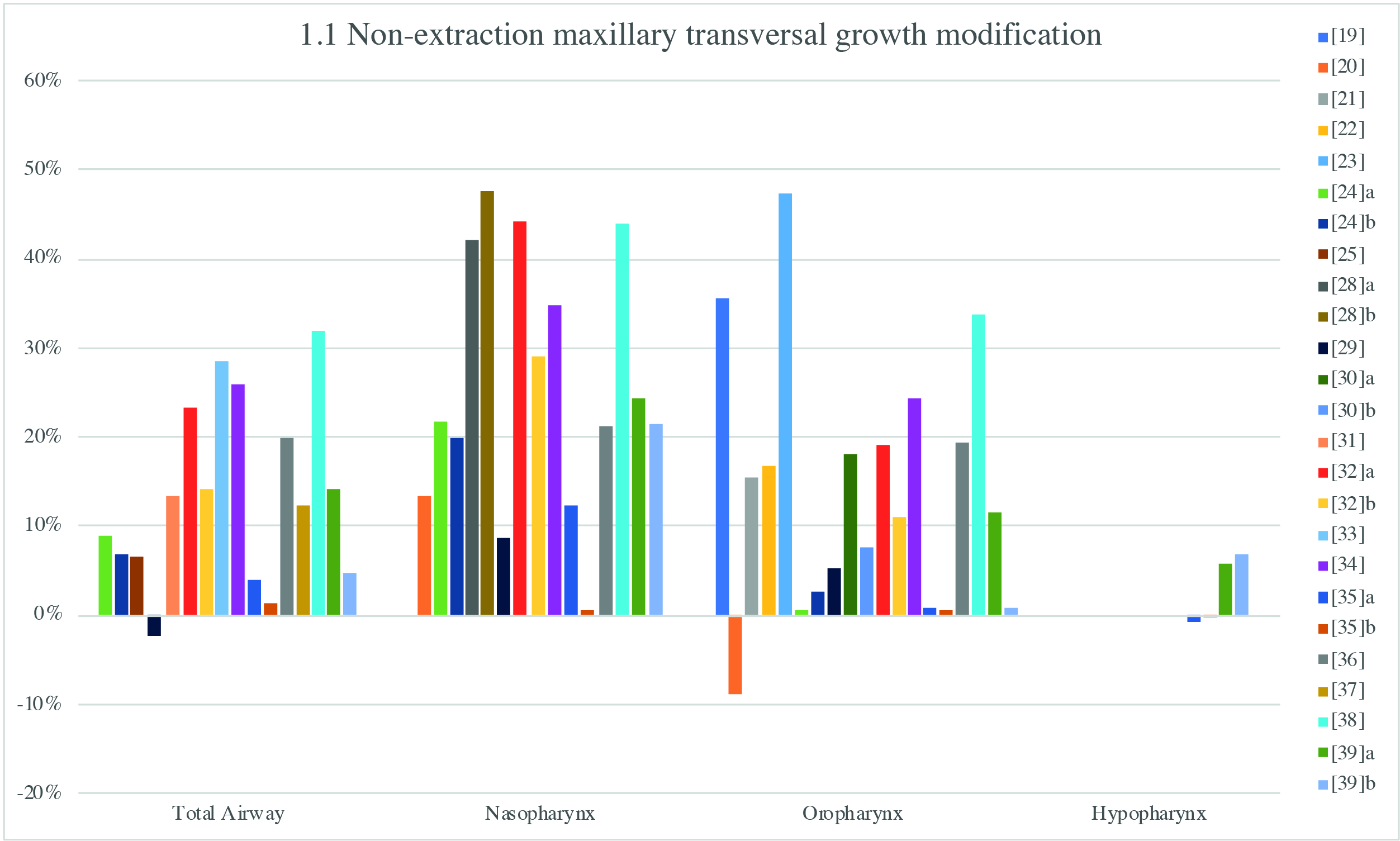

Supplement: Supplementary file 1 — Supplementary file1 (JPG 1501 KB) [file 784_2023_5207_MOESM1_ESM.jpg]

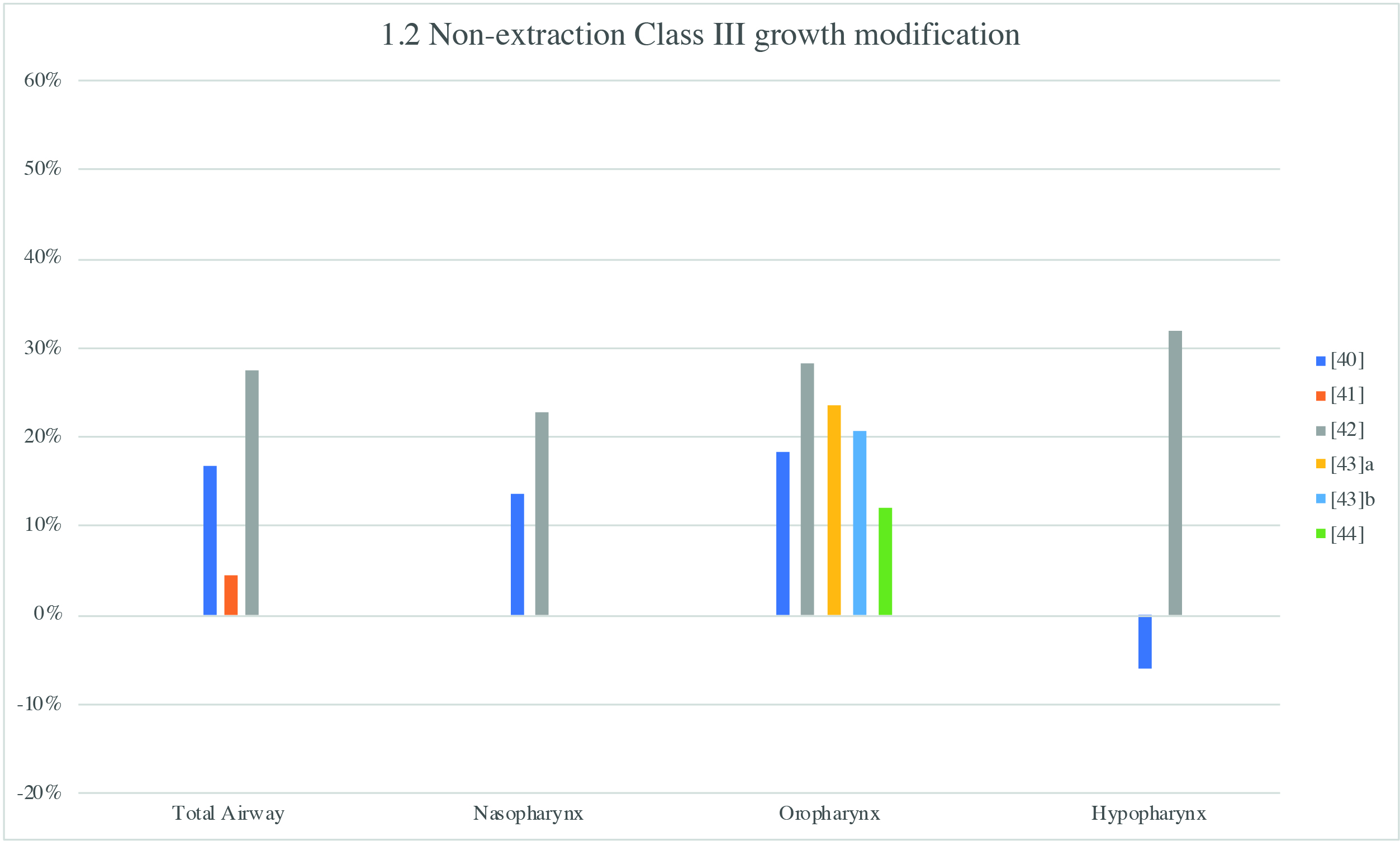

Supplement: Supplementary file 2 — Supplementary file2 (JPG 1221 KB) [file 784_2023_5207_MOESM2_ESM.jpg]

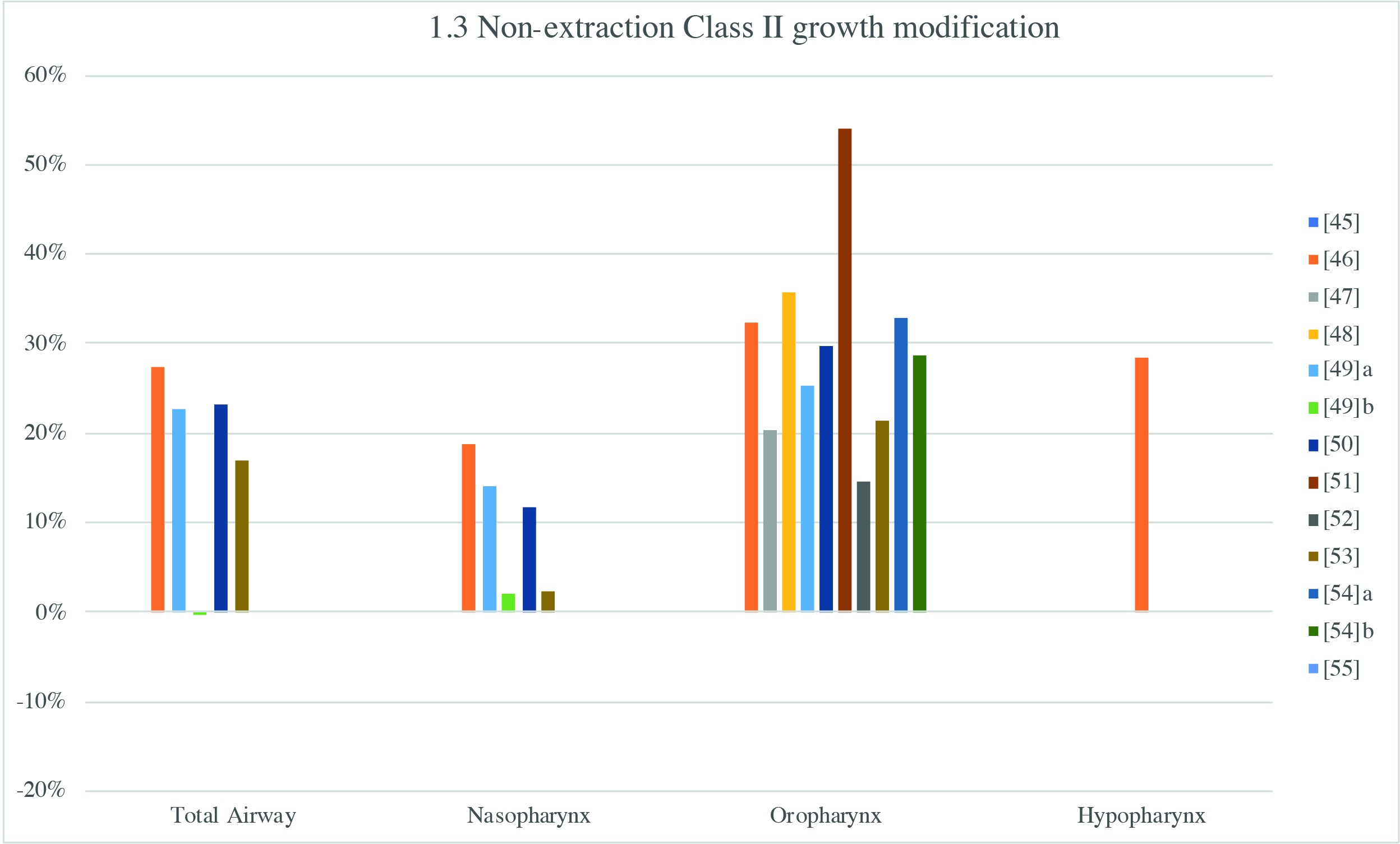

Supplement: Supplementary file 3 — Supplementary file3 (JPG 1327 KB) [file 784_2023_5207_MOESM3_ESM.jpg]

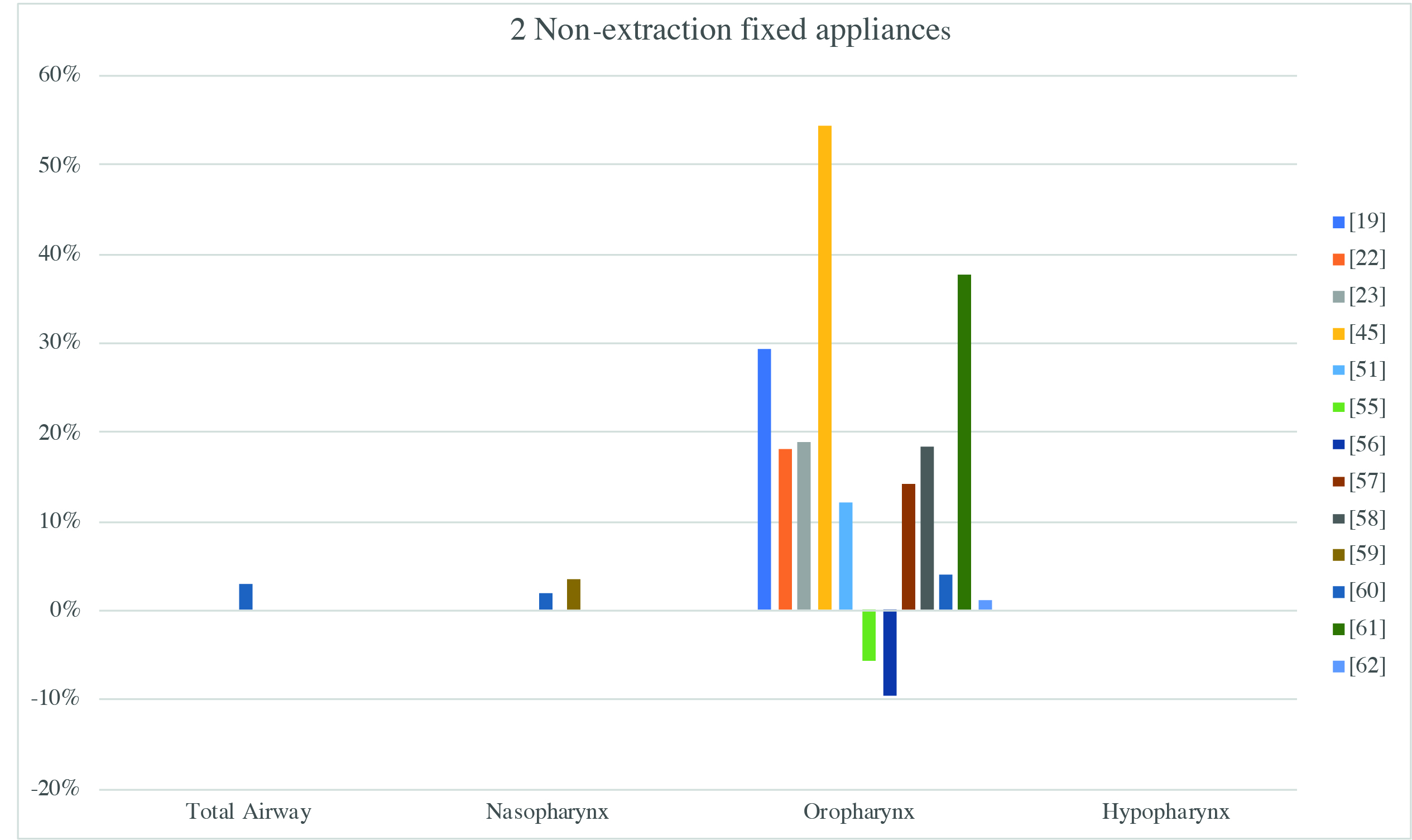

Supplement: Supplementary file 4 — Supplementary file4 (JPG 1280 KB) [file 784_2023_5207_MOESM4_ESM.jpg]

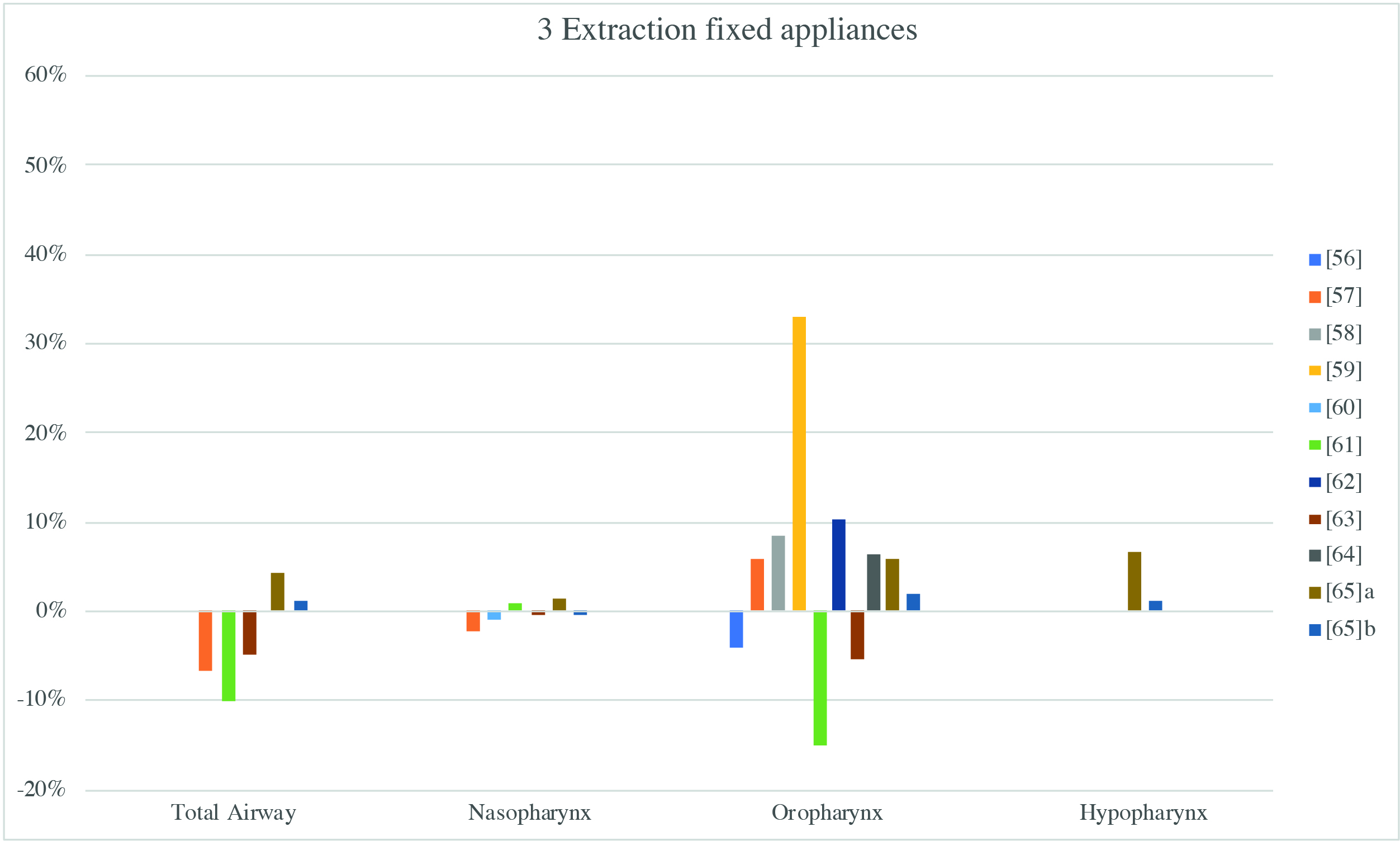

Supplement: Supplementary file 5 — Supplementary file5 (JPG 1254 KB) [file 784_2023_5207_MOESM5_ESM.jpg]
